# Enhanced Deep Neural Network for Prostate Segmentation in Micro-Ultrasound Images

**DOI:** 10.3390/s25226815

**Published:** 2025-11-07

**Authors:** Ahmed AL-Qurri, Asem Thaher, Mohamed Khaled Almekkawy

**Affiliations:** 1 The School of Electrical Engineering and Computer Science, Pennsylvania State University, University Park, PA 16802, USA; aqa6122@psu.edu; 2 Independent Researcher, State College, PA 16803, USA

**Keywords:** UNet, UNet++, Transformer, CNN, attention, medical imaging, Micro-Ultrasound, Hypergraph Neural Network, Mamba

## Abstract

Prostate cancer is a global health concern, and early diagnosis plays a vital role in improving the survival rate. Accurate segmentation is a key step in the automated diagnosis of prostate cancer; however, manual segmentation remains time-consuming and challenging. Micro-Ultrasound (US) is particularly well-suited for prostate cancer detection, offering real-time imaging with a resolution comparable to that of MRI. This enables improved spatial resolution and detailed visualization of small anatomical structures. With recent advances in deep learning for medical image segmentation, precise prostate segmentation has become critical for biopsy guidance, disease diagnosis, and follow-up. However, segmentation of the prostate in micro-US images remains challenging due to indistinct boundaries between the prostate and surrounding tissue. In this work, we propose a model for precise micro-ultrasound image segmentation. The model employs a dual-encoder architecture that integrates Convolutional Neural Networks (CNN) and Transformer-based encoders in parallel, combined with a fusion module to capture both global dependencies and low-level spatial details. More importantly, we introduce a decoder based on Mamba v2 to enhance segmentation accuracy. A Hypergraph Neural Network (HGNN) is employed as a bridge between the dual encoders and Mamba decoder to model correlations among non-pairwise connections. Experimental results on micro-US datasets demonstrated that our model achieved superior or comparable performance to state-of-the-art methods, with a Dice score of 0.9416 and an HD95 of 1.93.

## 1. Introduction

Prostate cancer is the second most prevalent cancer worldwide, with over 1.2 million new cases reported in 2020 [1]. Accurate imaging is critical for an early diagnosis. Traditionally, lesions in the prostate are identified using multiparametric MRI (mpMRI), which provides high-resolution anatomical and functional information about the lesions. However, MRI is expensive, time-consuming, and often inaccessible in many clinical settings, limiting its widespread adoption [2]. Ultrasound has become a widely adopted imaging modality owing to its low cost and high accessibility [3]. Micro-Ultrasound (micro-US) has recently emerged as a promising alternative, operating at substantially higher frequencies (typically 29 MHz or greater) than conventional ultrasound, thereby offering improved spatial resolution for prostate imaging [4]. Medical image segmentation plays a critical role in clinical practice by enabling the automatic localization of the prostate capsule. Although manual segmentation is feasible, it remains a time-consuming and labor-intensive task that is often inadequate for capturing the broader pathological context [5,6,7]. Recently, deep learning has been successfully applied to medical image segmentation. Nevertheless, despite advances in neural network architectures, segmentation remains a challenging task owing to noisy and scattered features, low resolution, weak boundaries, and irregular shapes of human organs [6,8,9].

In deep learning architectures, the convolution operation is the fundamental building block of U-Net [10,11]; however, its inherently limited receptive field constrains the network’s ability to capture global context [5]. To overcome this limitation, attention mechanisms have been introduced into deep learning. Attention allocates computational resources to regions containing the most relevant information, mimicking the functionality of the human visual system [6,12]. Consequently, several attention mechanisms have been proposed that can be categorized according to various criteria. One common categorization is based on the dimensions along which the attention feature map functions. For instance, channel attention exploits inter-channel dependencies, which are employed in Squeeze-and-Excitation (SE) networks [12]. In contrast, spatial attention emphasizes regions within feature maps that contain critical information while suppressing less relevant areas, as demonstrated in Non-Local Neural Networks [13]. Attention mechanisms can also be categorized based on the operation of the attention map [5]. For example, global attention enhances interactions across both channels and spatial locations, as in DANet [5,14], whereas local attention, such as in CBAM [15], focuses on specific spatial regions and their relationships with the channels.

Recently, Transformer-based models have gained prominence, initially introduced for sequence-to-sequence tasks in natural language processing (NLP) [16]. Transformers have been adopted across various domains due to their powerful attention mechanisms. Unlike CNN-based models, they overcome the limitation of restricted receptive fields by employing a self-attention (SA) mechanism [17]. The SA captures the internal correlations among all input tokens, enabling the modeling of long-range dependencies [18]. The Transformer architecture incorporates multiple self-attention blocks, known as multi-head self-attention (MHSA), which operate in parallel to generate diverse feature representations [17,18]. Building on this, Dosovitskiy et al. [19] introduced the Vision Transformer (ViT) for computer vision tasks, such as image classification, achieving state-of-the-art results on ImageNet. Moreover, Cao et al. [20] introduced Swin-Unet, the first U-shaped segmentation network based entirely on a Transformer architecture [21]. Swin-Unet integrates Swin Transformer blocks, where Window Multi-Head Self-Attention (W-MSA) captures fine-grained details within each window, and Shifted Window Self-Attention (SW-MSA) models cross-window interactions [22]. Unlike U-Net, Swin-Unet replaces conventional upsampling with patch-expanding layers, eliminating the need for convolutions or interpolation [6,23,24]. Nevertheless, despite its strong capability to capture the global context, the Transformer remains limited in modeling fine-grained details due to its lack of spatial inductive bias for local information. This limitation is particularly evident in medical image segmentation tasks [25].

To leverage the strengths of both Transformers and U-Net, Chen et al. [26] proposed the TransUNet. This hybrid architecture employs Convolutional Neural Networks (CNNs) to extract low-level features and a Transformer to capture global information [25]. Furthermore, hybrid models, such as TransFuse, adopt parallel encoder branches. In TransFuse [25], both a CNN-based spatial branch and a transformer-based global branch are fused using a BiFusion module [24,27]. In the BiFusion module, Transformer features are refined using a channel attention SE block [12], whereas the CNN features are enhanced using a spatial attention module inspired by CBAM [15]. The features from both branches were then combined using a Hadamard product to model their interactions. Similarly, CoTrFuse [28] follows a dual-branch strategy but incorporates Swin Transformer blocks for the global branch and EfficientNet blocks for the spatial branch, with feature fusion achieved through a specialized STCF module. Other researchers have focused on improving decoder performance. For instance, TransNorm [29] employs spatial normalization outputs from the Transformer to construct a two-level attention gate, where channel attention normalizes the feature representation to emphasize more informative channels and Transformer-produced spatial coefficients to amplify the relevant areas of the feature map. Other architectures, such as MS-TransUNet++ [30] and CoT-UNet++ [31], enhance CNN-Transformer hybrid architectures by introducing dense skip connections between encoders and decoders at multiple levels, similar to U-Net++ [6]. Some works have explored the frequency domain. For example, Discrete Fourier Transform (DFT) modules have been integrated to capture long-range dependencies more effectively [32]. Another line of research utilized deep supervision, which was first introduced in Google’s Inception architecture [5,33]. U-Net++ adopts deep supervision to help intermediate layers learn discriminative features and mitigate vanishing gradients [34]. Other network architectures incorporate side outputs for deep supervision. For example, BASNet [35] employs deep supervision via side outputs and was proposed for salient-object detection. One network relevant to this research is MicroSegNet, which employs multi-scale deep supervision for prostate segmentation from micro-ultrasound images [4].

In this paper, we propose an enhanced network architecture that utilizes a dual encoder, comprising both CNN and Transformer encoders, to effectively capture both local and global contextual information. The decoder is designed based on Mamba v2 in combination with CNN layers to improve the segmentation accuracy. Additionally, a Hypergraph Neural Network (HGNN) is integrated into the skip connections to capture non-pairwise correlations. To mitigate overfitting, we employed a tailored ultrasound-specific augmentation scheme incorporating depth attenuation, Gaussian shadows, haze artifacts, and speckle reduction.The experimental results demonstrate that the proposed architecture outperforms state-of-the-art segmentation models, achieving a superior segmentation accuracy. Our contributions can be summarized as follows:

- We propose a model for precise Micro-Ultrasound medical image segmentation composed of dual encoders with a fusion module designed to capture both local details and long-range dependencies. A Hypergraph Neural Network (HGNN) is integrated into the skip connections to model non-pairwise correlations.
- To further enhance segmentation accuracy, a Mamba-based decoder is incorporated, utilizing VSSD blocks built upon Mamba-2 and NC-SSD.
- Experimental results demonstrate that our method achieves superior performance on the Micro-Ultrasound (US) prostate medical image segmentation dataset.

## 2. Materials and Methods

### 2.1. Overall Architecture

**Figure 1:**
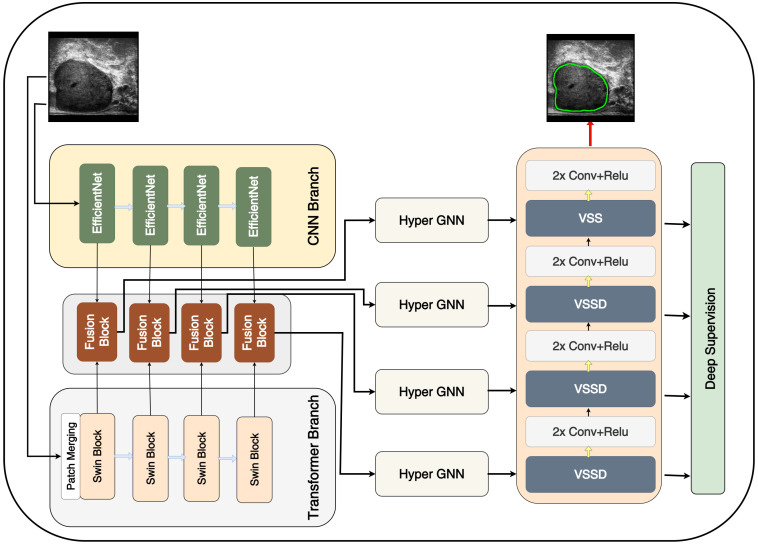
Overall network architecture with dual encoders, Hyper GNN, and Mamba decoder.

Inspired by CoTrFuse [28], we designed a network comprising two parallel branches. One branch employs a Transformer (Swin blocks) to capture the global context, whereas the other branch utilizes EfficientNet blocks to extract local details. The outputs from both branches are fused using a module based on BiFusion [25]. The overall architecture of the model is illustrated in Figure 1. On the decoder side, a combination of CNN layers and Visual State Space Duality (VSSD) blocks based on Mamba v2 was employed. The VSSD incorporates Non-Causal SSD (NC-SSD) modules [36] to enhance feature representation. Furthermore, inspired by BASNet [35], we integrated side outputs from the decoder for deep supervision, leveraging complementary information across intermediate prediction maps [6]. These outputs were upsampled and incorporated into the training to provide guidance at multiple levels. This approach accelerates convergence and mitigates the vanishing gradient problems. A more detailed discussion of the network architecture is provided in the following section.

#### 2.1.1. Dual Encoder

**Figure 2:**
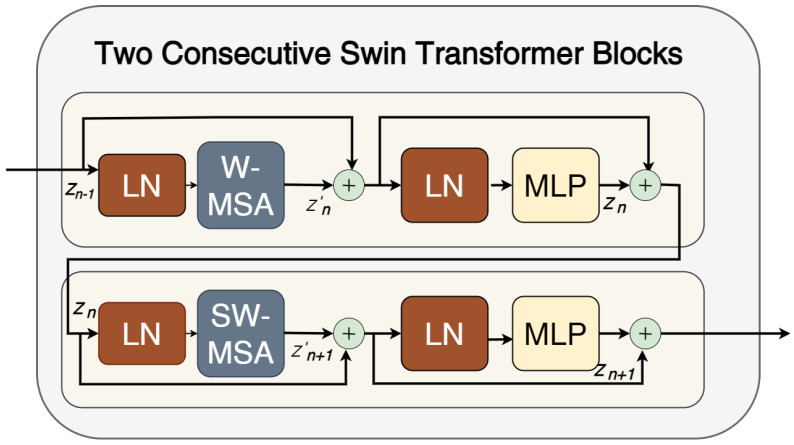
Diagram of the Swin-Transformer Block based on Swin-Unet. The Swin transformer employs a window-based multi-head self-attention (W-MSA) module and a shifted window-based multi-head self-attention (SW-MSA) module.

The dual-encoder architecture employs EfficientNet to extract local features [37]. EfficientNet was developed using a neural architecture search (NAS) to systematically scale convolutional networks, resulting in a family of highly efficient models that balance network width, depth, and resolution. Each EfficientNet model is constructed from a series of Inverted Residual Blocks, also referred to as MBConv blocks [38]. The other branch employs Swin Transformer blocks with patch-merging layers, similar to [20,28]. As mentioned in the introduction section, Cao et al. [20] introduced Swin-Unet, a Transformer-based architecture that incorporates a hierarchical structure and a window-shifting mechanism to enhance feature representation. Swin Transformer blocks perform feature learning through a window-based multi-head self-attention (W-MSA) module, followed by a shifted window multi-head self-attention (SW-MSA) module [20], as illustrated in Figure 2. Specifically, the W-MSA captures the local dependencies among pixels within each window, whereas the SW-MSA models the global interactions across windows, thereby enabling cross-contextual attention for feature recalibration. The mathematical formulation of the Swin Transformer blocks is shown in Equations (1)–(4):

(1) $$Z_{n}^{\prime }=\mathrm{WMSA}\left(LN\left(Z_{n-1}\right)\right)+Z_{n-1}$$

(2) $$Z_{n}=\mathrm{MLP}\left(LN\left(Z_{n}^{\prime }\right)\right)+Z_{n}^{\prime }$$

(3) $$Z_{n+1}^{\prime }=\mathrm{SWMSA}\left(LN\left(Z_{n}\right)\right)+Z_{n}$$

(4) $$Z_{n+1}=\mathrm{MLP}\left(LN\left(Z_{n+1}^{\prime }\right)\right)+Z_{n+1}^{\prime }$$

where $Z_{n}^{\prime }$ and $Z_{n}$ represent the outputs of the WMSA/SWMSA module and the MLP module of the $n^{th}$ block.

The self-attention (SA) mechanism is the core component of the Transformer’s block, as shown in Equation (5):

(5) $$Attention\left(Q,K,V\right)=softmax\left(\frac{QK^{T}}{\sqrt{d}}+B\right)V$$

where $Q,K,V\in R^{P^{2}\times \;d}$ represent the query, key, and value matrices, respectively. $P^{2}$ and d donate the number of patches in a window and the dimension of either the query and key, respectively. B is a relative position bias, and it is based on the bias matrix $B^{\prime }\in R^{\left(2P-1)\times (2P+1\right)}$.

Similar to Swin-Unet, the first step in the Swin Transformer branch of our model is a linear embedding layer that projects the input features into an arbitrary dimension. This is followed by a patch-merging layer that performs downsampling while increasing the feature dimension before the Transformer blocks process the data. Skip connections carrying multi-scale features from the Transformer encoder are later fused with the feature maps from the CNN encoder in the fusion module, as discussed in the subsequent section. For simplicity, the linear embedding layer is omitted from Figure 1.

The two branches are merged using a fusion module as illustrated in the next section.

#### 2.1.2. Fusion Module

TransFuse [25] and CoTrFuse [28] both leverage dual encoders that are combined through fusion modules. Although the two fusion modules share similar concepts, they differ in their implementations. Both modules incorporate channel and spatial attention mechanisms to enhance feature representation.

For example, the BiFusion module [25] in TransFuse uses an SE block [12] as channel attention to emphasize global information from the Transformer branch, while a CBAM block [15] is used as spatial attention to capture fine-grained details from the CNN branch.

**Figure 3:**
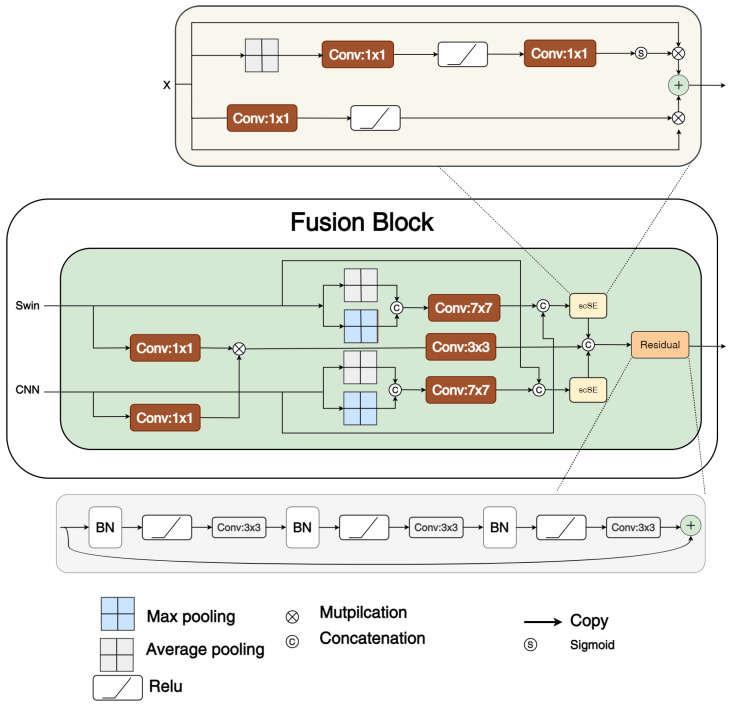
Architecture of the proposed fusion module block, adapted from the STCF fusion module [28]. The SE block and CBAM are used to perform channel and spatial attention, respectively.

The STCF fusion module in CoTrFuse adopts a similar attention-based approach. However, unlike BiFusion, where channel attention is applied to the Transformer feature map and spatial attention is applied to the CNN feature map, STCF applies both attention mechanisms to both feature maps before summing them. In our model, we adopted the STCF fusion strategy, as shown in Figure 3.

#### 2.1.3. Hyper GNN

In an ordinary graph, each edge connects only two nodes, representing the pairwise correlations. In contrast, a hypergraph employs hyperedges that can connect more than two nodes, thereby enabling the modeling of non-pairwise correlations. This makes hypergraphs more effective in capturing complex relationships. Han et al. [39] introduced a Vision Hypergraph Neural Network (ViHGNN). Subsequently, Feng et al. [40] proposed a Hypergraph Neural Network (HGNN), which constructs a hypergraph from an image using K-Nearest Neighbors (KNN). In the context of medical image segmentation, Peng et al. [41] integrated an HGNN into a U-Net for MRI segmentation. More recent work has focused on adaptive hypergraph construction. For example, unlike earlier methods that relied on a fixed number of neighbors per node for graph construction, Chai et al. [42] proposed an adaptive strategy for hyperedge formation known as Adaptive Hypergraph Construction. This method utilizes the K-Nearest Neighbors (KNN) algorithm to generate a matrix used to compute the degree of each node, which in turn guides the construction of hyperedges. Readers are referred to [42] for further details regarding the Adaptive Hypergraph Construction procedure. Their Adaptive Hypergraph Construction method models the shape attributes more accurately, particularly in medical imaging. Furthermore, Chai et al. extended the concept of convolutional sliding windows to include hypergraphs. In this approach, the sliding-window-based convolution mechanism employs fixed-size kernels to convolve the feature maps, enabling interaction with local neighboring pixels across the entire image through stride operations.

**Figure 4:**
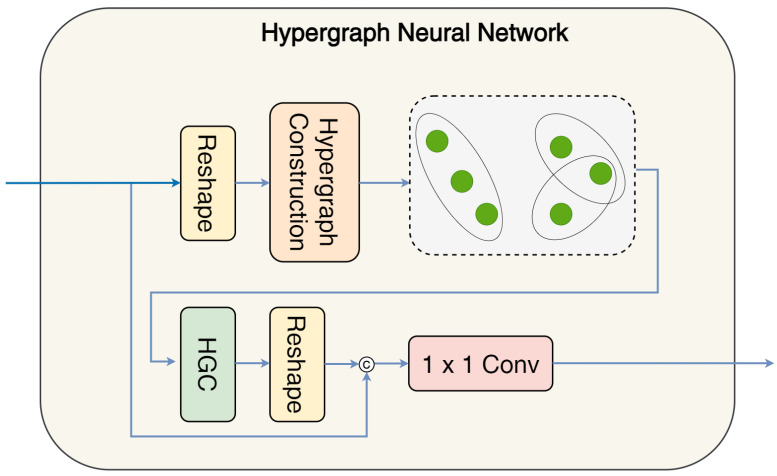
The Adaptive Hypergraph Construction module, implemented following the method proposed by Chai et al. [42]. This module constructs hyper-edges adaptively based on node degree, utilizing the K-Nearest Neighbors (KNN) algorithm to form the adjacency matrix for hypergraph generation.

The Adaptive Hypergraph Construction is illustrated in Figure 4, with hypergraph convolution denoted as HGC.

#### 2.1.4. Mamba Decoder and VSSD Block

**Figure 5:**
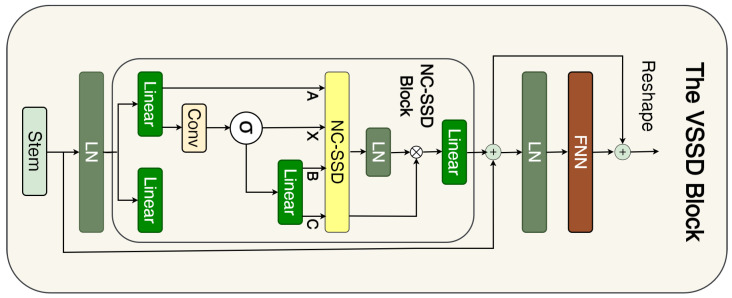
The VSSD block based on [45] that employ Mamba2.

Motivated by the success of Transformers in capturing long-range dependencies, Mamba with State Space Models (SSMs) has been proposed to capture the global context while avoiding the high computational cost of Vision Transformers (ViTs). The Mamba S6 model improves upon the S4 model by introducing a selective mechanism and hardware optimization. To adapt Mamba for computer vision, VMamba [36] converts non-causal visual images into sequences of ordered patches using a Cross-Scan Module (CSM). The S6 block was further enhanced in Mamba2 [43] through the introduction of State Space Duality (SSD) [44]. Recently, Xu et al. proposed a Non-Causal SSD (NC-SSD) that eliminates the need for a causal mask and specialized scanning paths. Building on this, they introduced Visual State Space Duality (VSSD) to replace the VSS vision block [45]. The VSSD block based on Mamba2 is shown in Figure 5.

The VSSD block exhibits a design similar to that of the Mamba block [46]; however, it employs the NC-SSD module instead of the standard Mamba SSD. Vision Mamba models, such as Vision Mamba [47] and VMamba [36] typically rely on image traversal mechanisms that use different scanning routes to flatten a 2D image into a 1D sequence. In contrast, NC-SSD introduces an enhanced algorithm by leveraging the fact that the matrix A in Mamba 2 [43] is reduced to a scalar [45]. This design eliminates the need for a specific scanning route and removes causal mask requirements. For more detailed information on the NC-SSD, readers are referred to [45]. As shown in Figure 1, our decoder employs four VSSD blocks, with convolution and ReLU activation layers inserted between them, each of which is repeated twice. The configuration of the VSSD blocks is as follows: channel dimensions [256, 384, 448, 480] and number of heads [2, 4, 8, 16]. At each stage, each block was applied once without repetition.

### 2.2. Dataset

For this study, we utilized a prostate segmentation dataset based on micro-ultrasound (micro-US) images from [4]. The dataset is publicly available at https://zenodo.org/records/10475293 and was accessed on 2 February 2025. To the best of our knowledge, this is the only publicly available micro-US dataset. Micro-ultrasound is a 29-MHz imaging technology that provides a resolution that is 3–4 times higher than that of conventional ultrasound. A key advantage of micro-US over other modalities, such as MRI and standard ultrasound, is its real-time visualization capability, eliminating the need for image fusion, which can account for up to 50% of diagnostic errors. The dataset was collected from 75 men who underwent micro-US-guided prostate biopsy at the University of Florida [4]. Each patient underwent a prostate scan from left to right, capturing approximately 200–300 micro-US images. The images were converted from B-mode to a DICOM series with embedded pixel spacing information. Ground-truth prostate annotations were performed by two non-expert annotators and one expert annotator for all 75 patients. Additionally, for evaluation purposes, three annotators manually segmented the prostate capsule in 20 test cases. For model training, 2060 micro-US images from 55 patients were used, and 758 images from 20 patients were reserved for testing. The dataset collection study was approved by the Institutional Review Board at the University of Florida.

### 2.3. Augmentation

Ultrasound-specific data augmentation techniques were employed to prevent overfitting. Four different augmentations have been utilized: Depth Attenuation, Gaussian Shadow, Haze Artifact, and Speckle Reduction using the USAugment (version 1.0.1) [48] (https://github.com/adamtupper/ultrasound-augmentation, access date (7 May 2025)). Depth Attenuation simulates the gradual loss of ultrasound wave energy as it propagates through the tissue, causing the intensity to decrease with increasing distance from the probe. Haze Artifact models semi-static noise bands that occasionally appear in the ultrasound images. Gaussian shadows replicate the acoustic shadows caused by air or tissue obstructing wave propagation, generating two-dimensional Gaussian-shaped shadows with randomly selected parameters. Speckle noise, arising from interference among ultrasound waves, is mitigated using Speckle Reduction, which applies a bilateral filter with randomly sampled parameters to reduce speckle patterns. Empirically, we found that applying these augmentations with a probability of 0.2 yields the best result.

### 2.4. Loss Function and Evaluation

Following [4], the AG-BCE loss function was employed. The AG-BCE loss is based on the BCE loss but accounts for the characteristics of prostate segmentation in micro-US images by penalizing prediction errors more heavily in challenging regions, particularly along the borders between the prostate and bladder. Hence, it assigns different weights to each pixel.

(6) $$L_{\mathrm{AG}-\mathrm{BCE}}=-\frac{1}{N}\sum_{i=1}^{N}w_{i}\left[y_{i}\log\left(p_{i}\right)+\left(1-y_{i}\right)\log\left(1-p_{i}\right)\right]$$

where

- *N* is the total number of pixels,
- $w_{i}\in \left\{0,1\right\}$ is the is the weight of assigned to pixel *i*,
- $y_{i}\in \left\{0,1\right\}$ is the ground truth label for pixel *i*,
- $p_{i}\in \left[0,1\right]$ is the predicted probability for pixel *i*.

The AG-BCE loss function differentiates between hard and easy regions. Hard regions are defined as areas where expert and non-expert annotations disagree, whereas easy regions are defined as those where both annotations coincide. AG-BCE assigns a weight (denoted by Equation (6) as *w* of four hard regions and one easy region, following the implementation in MicroSegNet [4]. For a more detailed explanation of the AG-BCE loss formulation, readers are referred to [4].

The average Dice Similarity Coefficient (DSC) and average Hausdorff Distance (HD95) were used as evaluation metrics. HD95 measures the Hausdorff distance by considering the 95th percentile of the distances between the boundary points of the two sets. Unlike the standard Hausdorff distance, which reports the maximum distance, HD95 reduces the influence of outliers by ignoring the extreme values [6]. The Dice Similarity Coefficient (DSC) is defined as

(7) $$\mathrm{DSC}\left(G,P\right)=\frac{2\;|G\cap P|}{\left|G|+|P\right|},$$

where *G* denotes the set of ground-truth pixels, and *P* denotes the set of predicted pixels. The Hausdorff distance between two sets *G* and *P* is defined as

(8) $$d_{H}\left(G,P\right)=\max\left\{d_{h}\left(G,P\right),\;d_{h}\left(P,G\right)\right\},$$

where

(9) $$d_{h}\left(G,P\right)=\max_{g\in G}\;\min_{p\in P}∥g-p∥,$$

and

(10) $$d_{h}\left(P,G\right)=\max_{p\in P}\;\min_{g\in G}∥p-g∥.$$

Here, *G* and *P* denote the ground truth and the predicted outcome, respectively, while ∥·∥ represents the Euclidean distance. Intuitively, $d_{h}\left(G,P\right)$ is the maximum distance from any point in *G* to its nearest neighbor in *P*, and $d_{h}\left(P,G\right)$ is defined analogously.

In order to account for image resizing, the HD95 value is adjusted by multiplying it with a spacing parameter, which was specified as 0.033586 in the implementation described by [4].

### 2.5. Implementation Details

As a preprocessing step, all images were resized to 224×224. Following the setup in [4], training was conducted using an image patch size of 16 and a batch size of eight. Stochastic Gradient Descent (SGD) optimizer and momentum, with a decaying learning rate, is used as model optimizer. The initial learning rate, momentum, and weight decay were set to 0.01, 0.9, and 0.0001, respectively. An adaptive learning rate strategy was employed, which reduced the learning rate after a certain number of iterations. The number of epochs was set to 150. Both training and testing were implemented and conducted using PyTorch (Version: 2.7.1+cu118) on an NVIDIA RTX A4500 GPU with 20 GB of memory and Python 3.12.7.

## 3. Results and Discussion

Table 1 presents a comparison between our proposed model and the state-of-the-art (SOTA) methods, with Dice Similarity Coefficient (DSC) and 95th percentile Hausdorff Distance (HD95) used as evaluation metrics. The results in Table 1 represent the average performance across several runs. Our reported results are based on our own training, which was conducted for both 150 and 10 epochs to ensure a fair comparison. The only exception is the MicroSegNet scores, that is based on 10 epochs. This score is reported from [4]. As noted by H. Jiang et al. [4], MicroSegNet training was limited to 10 epochs to avoid overfitting. In contrast, overfitting in our model was mitigated through data augmentation, as discussed earlier. However, to ensure a fair comparison under the same conditions (with augmentation), Table 2 presents results for our model and an augmented version of MicroSegNet. As shown in Table 1, our model achieved its best performance when trained for 150 epochs. For comparison, we incorporated several models, some of which are recent, such as RWKV-UNet [49] and SegU-KAN [50]. For all evaluated models, we used AG-BCE as the loss function, similar to [4], to focus more on challenging regions over easily segmented ones during training.

We employed pretraining for certain models, such as Swin-UNet [20], but not all models in Table 1 were pretrained. As shown in Table 1, our model achieved the highest performance, consistently outperforming the competing approaches. Specifically, it attained an average Dice Similarity Coefficient (DSC) of 94.16% and a Hausdorff Distance (HD95) of 1.93 mm. For example, compared with Swin-UNet, the average DSC increased from 0.932 to 0.9416, corresponding to an improvement of nearly 1%, whereas the HD95 decreased from 2.04 mm to 1.93 mm, representing a reduction of approximately 5%. These experimental results demonstrate that the proposed network architecture outperforms state-of-the-art segmentation models, achieving a significantly improved segmentation accuracy. Moreover, Table 1 also reports the number of FLOPs, model parameters, GPU memory usage, and inference time for each model. Notice, the number of FLOPs and the number of parameters are calculated using the *calflops* library (https://github.com/MrYxJ/calculate-flops.pytorch, access date (11 March 2024)).

**Figure 6:**
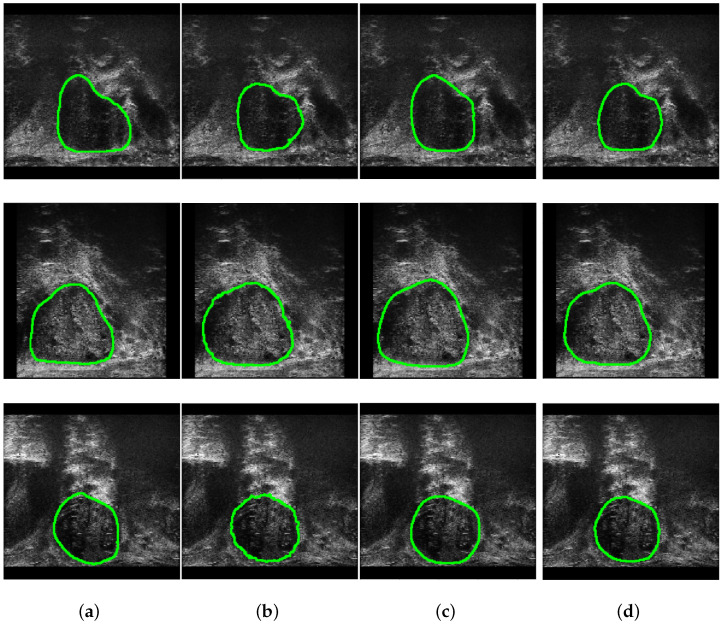
Qualitative comparison of CT slices from the Micro-US dataset, showing prostate boundaries donated as a green circle. From left to right: (**a**) Ground Truth, (**b**) SwinUNet, (**c**) MicroSegNet, and (**d**) our model’s prediction.

**Figure 7:**
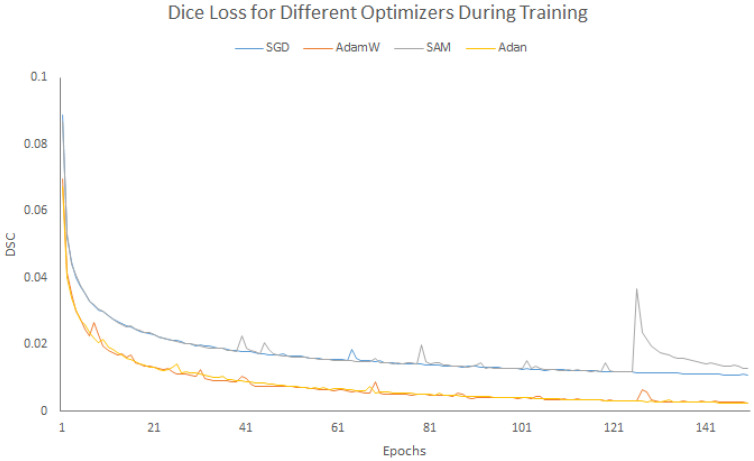
Dice Loss for different optimizers during training.

**Figure 8:**
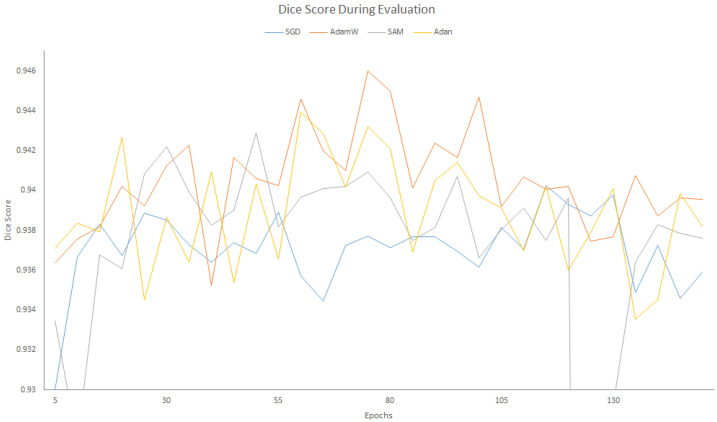
Dice Score During Evaluation.

Figure 6 presents the segmentation outcomes of the various approaches, namely, SwinUNet, MicroSegNet and our model on the Micro-ultrasound dataset from three images for qualitative comparison. To evaluate the impact of different optimizers, we conducted experiments on the Micro-Ultrasound dataset. Figure 7 and Figure 8 illustrate the results for four optimizers: SGD [57,58], AdamW [59], SAM [60,61], and Adan [62]. For clarity, the training losses and validation scores are presented in separate figures. SGD, which we used for our model, updates the gradient in each iteration using a randomly selected sample rather than computing the exact gradient. The AdamW optimizer is similar to Adam, but while the Adam optimizer adaptively adjusts the learning rate for each parameter based on first and second order moment estimates of the gradient [6] using the geometry curvature of the objective function [62], AdamW decouples the weight decay from the gradient update.

Sharpness-Aware Minimization (SAM) attempts to find parameter neighborhoods that have uniformly low loss, making it more robust to noisy labels in the training set by perturbing the loss landscape. SAM is particularly beneficial when applied to Vision Transformers (ViTs), as these architectures are more prone to end up in local minima than CNN-based architectures [63,64]. However, SAM requires forward and backward passes twice for each iteration. Adaptive Nesterov Momentum (Adan) introduces a new Nesterov Momentum Estimation (NME) method based on Nesterov acceleration. Its efficiency arises from the elimination of the overhead of computing the gradient at the extrapolation point. Figure 7 illustrates the behavior of each optimizer during the training. Both AdamW and Adan exhibited lower loss curves, indicating better performance than SGD and SAM. This trend is also confirmed by the evaluation curves shown in Figure 8. Unlike in Figure 7, the curves in Figure 8 are intertwined; however, careful examination reveals that AdamW and Adan show better accuracy than the others.

**Figure 9:**
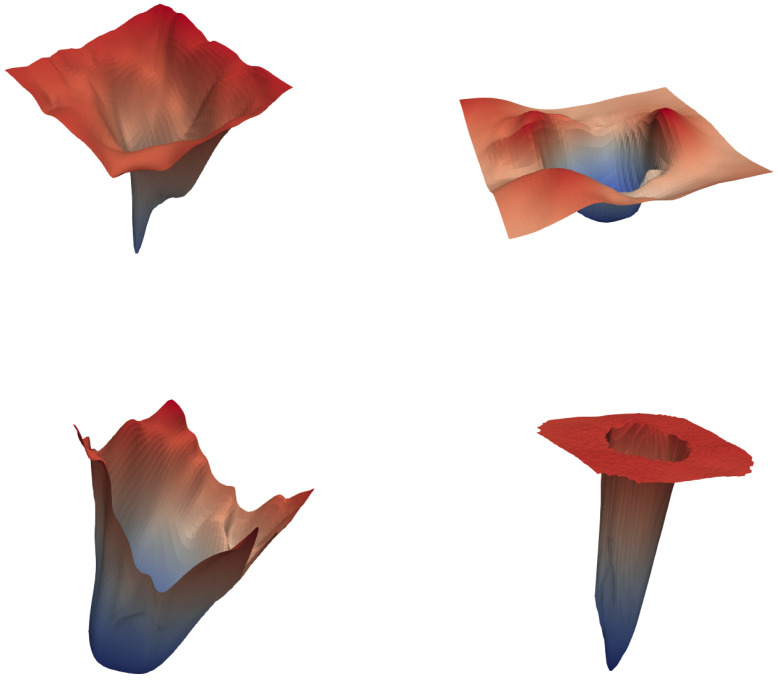
Loss surface visualization of different neural networks using the filter-normalization method. Top-left: UNet++; top-right: Swin-UNet; bottom-left: MicroSegNet; bottom-right: our model.

**Figure 10:**
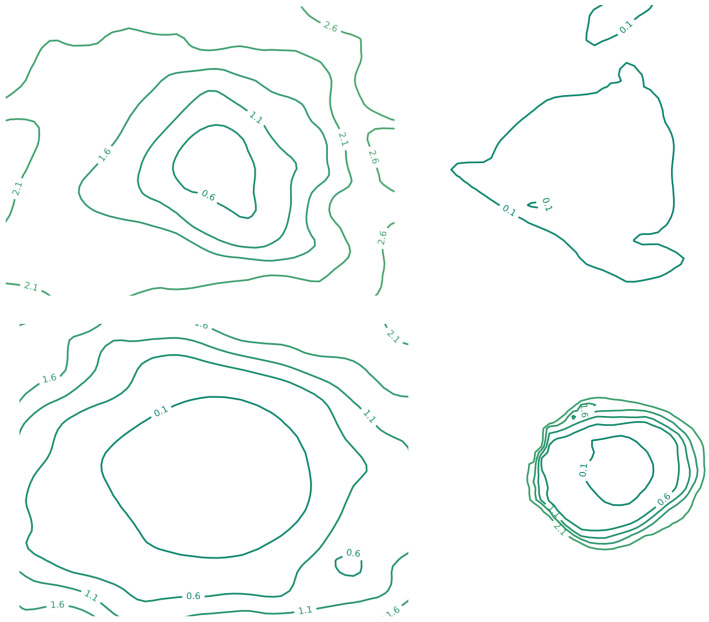
Contour maps of the 2D loss landscape for different neural networks using the filter-normalization method. Top left: U-Net++; top right: Swin-UNet; bottom left: MicroSegNet; bottom right: proposed model.

Figure 9 and Figure 10 depict the loss landscapes of different neural networks as 3D surface plots and contour maps, respectively. These visualizations were generated using the filter-normalization method [65], with the 3D surface renderings in Figure 9 produced using ParaView (Version 5.12) (http://paraview.org/, access date (5 March 2024)) software. The filter-normalization approach evaluates the loss landscape along filter-normalized directions, where the geometry of the landscape provides insights into model trainability and generalizability [64]. In particular, smoother and more convex landscapes are typically correlated with lower error values, whereas irregular or chaotic landscapes tend to yield higher training errors.

## 4. Ablation Study

Ablation studies were performed to assess the contribution of each component in the proposed method and to support the design decisions based on the performance outcomes of the Micro-Ultrasound dataset. These results reflect the average scores computed over multiple runs. The results for the five different configurations are summarized in Table 3. Notice that DS denotes deep supervision. Our analysis demonstrates the effectiveness of the proposed enhancements compared with the baseline model. As shown in the table, integrating the Mamba blocks into the decoder improved both the DSC and the 95th percentile HD95. The addition of deep supervision further enhanced the performance of both metrics. Table 3 also lists the number of FLOPs and parameters. It is noteworthy that the majority of components contribute minimally to the increase in FLOPs and parameters; the only exception is the integration of Mamba blocks within the decoder (VSSD), which leads to an increase in both metrics. Also, notice that adding the hyperGraph module slightly improved the DSC score, but it worsened the HD score. Hence, to verify the effectiveness of integrating HyperGraph, Table 4 presents a comparison of the model with and without HyperGraph. As shown, HyperGraph indeed enhances accuracy based on both metrics. Finally, the combination of Mamba, deep supervision, and ultrasound-specific data augmentation achieved the best average results for both HD95 and DSC scores.

Table 5 presents a comparison of the model performance based on different Swin block configurations. The Tiny (Swin-T) configuration has channel dimensions of [96, 192, 384, 768] and numbers of heads [3, 6, 12, 24], with the blocks repeated [2, 2, 6, 2]. The Small (Swin-S) configuration uses the same channel dimensions and numbers of heads but repeats the blocks [2, 2, 18, 2] [66]. Note that we did not test the larger Swin variants (Swin-B and Swin-L) due to their higher GPU memory requirements.

## 5. Conclusions

This work presents an improved model for ultrasound medical image segmentation, with particular emphasis on micro-US. The proposed architecture features a dual-encoder design: a Swin Transformer branch for capturing global context and a CNN-based branch for extracting fine local details. To further enhance feature representation, a hypergraph network is incorporated to model non-pairwise correlations. The decoder leverages the Mamba 2 architecture, built upon VSSD blocks, to improve localization accuracy. Furthermore, ultrasound-specific augmentation techniques, such as depth attenuation, were employed during training to mitigate overfitting and enhance robustness. Experimental results demonstrate that the proposed method achieves superior performance across multiple evaluation metrics, underscoring its potential to advance segmentation accuracy in ultrasound medical imaging. Future research could focus on mitigating the scarcity of Micro-Ultrasound (US) image datasets using semi-supervised or weakly supervised learning. These approaches can effectively leverage large volumes of unlabeled data to reduce annotation costs while preserving the segmentation boundary accuracy. In particular, weakly supervised methods may utilize alternative supervisory cues such as image-level tags, bounding boxes, or scribbles [67].

## Figures and Tables

**Table 1:** The segmentation accuracy on the micro-US dataset was evaluated using the average Dice score and 95% Hausdorff distance (HD95). The best results are indicated in bold. The Dice scores marked with * are reported in [4], and their values are provided with a precision of three decimal places. All results are based on training for 150 epochs and 10 epochs to ensure a fair comparison. FLOPs (G) denote the number of floating-point operations (in billions). #Params (M) indicates the number of parameters (in millions). GPU Mem (G) denotes GPU memory usage (in gigabytes). Inference (S) represents the inference time (in seconds).

Model	150 Epochs		10 Epochs					**Inference (S)**
	**DSC↑**	**HD↓**	**DSC↑**	**HD↓**	**FLOPs (G)**	**#Params (M)**	**GPU Mem (G)**	
Unet [26]	0.8897	5.94	0.8420	7.62	21.37	7.85	5.93	**120**
UNet++ [51]	0.9081	3.81 1	0.8894	4.82	53.1	9.16	11.39	134
TransUNet [26]	0.9293	2.39	0.9303	2.20	58.49	105.28	10.57	133
Swin-UNet [20]	0.9327	2.04	0.9218	2.49	17.4	41.38	19.46	165
TransNorm [29]	0.9214	2.63	0.9232	2.45	62.18	117.63	16.54	130
HiFormer-B [52,53]	0.8967	4.40	0.8784	5.34	8.045	25.51	11.40	141
CoTrFuse [28]	0.9266	2.74	0.9065	3.42	33.07	56.19	15.30	166
RWKV-UNet [49]	0.8866	4.62	0.7958	5.85	57.44	120.24	10.96	122
H-vmunet [54]	0.8817	3.34	0.8746	4.39	1140.6	8.97	**3.79**	167
Seg. U-KAN [50]	0.8918	5.09	0.8409	6.81	14.02	**6.35**	15.01	130
VM-UNet [55,56]	0.9042	2.89	0.8630	5.30	**7.56**	34.62	18.97	126
MicroSegNet [4]	0.9341	2.23	**0.939** *	**2.02**	58.49	105.28	10.96	131
**Ours**	**0.9416**	**1.93**	0.9380	**2.02**	70.72	93.13	11.93	202

**Table 2:** Quantitative comparison between our model and MicroSegNet when data augmentation is applied to MicroSegNet.

Methods	DSC↑	HD95↓
MicroSegNet with Augmentation (150 Epochs)	0.9321	2.29
MicroSegNet with Augmentation (10 Epochs)	0.9356	2.04
Ours	**0.9416**	**1.93**

**Table 3:** Contribution of each component to the overall performance on the Micro-Ultrasound dataset. FLOPs (G) denote the number of floating-point operations (in billions). #Params (M) indicates the number of parameters (in millions).

	Mamba (VSSD)	DS	HyperGraph	Augment.	FLOPs (G)	#Params (M)	DSC↑	HD↓
Baseline					**42.4**	**66.79**	92.84	2.28
	✓				68.5	93.05	93.03	2.19
	✓	✓			68.5	93.05	93.73	1.95
	✓	✓	✓		70.74	93.13	93.75	1.99
Proposed Model	✓	✓	✓	✓	70.72	93.13	**94.16**	**1.93**

**Table 4:** Comparison of the model with and without HyperGraph on the Micro-Ultrasound Dataset.

Methods	DSC↑	HD95↓
Without HyperGraph	93.96	1.95
With HyperGraph (Overall)	**94.16**	**1.93**

**Table 5:** Ablation study on the model scale for the Swin Blocks.

Methods	DSC↑	HD95↓
Swin-T	**94.16**	**1.93**
Swin-S	93.96	1.95

## Data Availability

The Micro-ultrasound (micro-US) images segmentation dataset is publicly available at https://zenodo.org/records/10475293 and was accessed on 2 February 2025.
